# Effect of Probiotic Supplementation on Newborn Birth Weight for Mother with Gestational Diabetes Mellitus or Overweight/Obesity: A Systematic Review and Meta-Analysis

**DOI:** 10.3390/nu12113477

**Published:** 2020-11-12

**Authors:** Chun-Chi Wang, Yu-Tang Tung, Hua-Ching Chang, Chang-Hsien Lin, Yang-Ching Chen

**Affiliations:** 1 Department of Family Medicine, Taipei Medical University Hospital, Taipei Medical University, Taipei City 110, Taiwan; chunchi_mei@hotmail.com (C.-C.W.); 862077@h.tmu.edu.tw (C.-H.L.); 2 Graduate Institute of Metabolism and Obesity Sciences, Taipei Medical University, Taipei City 110, Taiwan; f91625059@tmu.edu.tw; 3 Nutrition Research Center, Taipei Medical University Hospital, Taipei City 110, Taiwan; 4 Cell Physiology and Molecular Image Research Center, Wan Fang Hospital, Taipei Medical University, Taipei City, 110, Taiwan; 5 Department of Dermatology, Taipei Medical University Hospital, Taipei City 110, Taiwan; 163052@h.tmu.edu.tw; 6 School of Nutrition and Health Sciences, College of Nutrition, Taipei Medical University, Taipei City 110, Taiwan; 7 Department of Family Medicine, School of Medicine, College of Medicine, Taipei Medical University, Taipei City 110, Taiwan

**Keywords:** pregnancy, gut microbiota, probiotics, gestational diabetes mellitus (GDM), obesity, overweight, newborn birth weight

## Abstract

High birth weight indicates the future risk of obesity and increased fat mass in childhood. Maternal gestational diabetes mellitus (GDM) or overweight are powerful predictors of high birth weight. Studies on probiotic supplementation during pregnancy have reported its benefits in modulating gut microbiota composition and improving glucose and lipid metabolism in pregnant women. Therefore, probiotic intervention during pregnancy was proposed to interrupt the transmission of obesity from mothers to newborns. Thus, we performed a meta-analysis to investigate the effect of probiotic intervention in pregnant women with GDM or overweight on newborn birth weight. We searched PubMed, EMBASE, Cochrane Library, and Web of Science databases up to 18 December 2019. Randomized controlled trials (RCTs) comparing pregnant women with GDM or overweight who received probiotic intervention during pregnancy with those receiving placebo were eligible for the analysis. Newborn birth weights were pooled to calculate the mean difference with a 95% confidence interval (CI). Two reviewers assessed the trial quality and extracted data independently. Seven RCTs involving 1093 participants were included in the analysis. Compared with the placebo, probiotics had little effect on newborn birth weight of pregnant women with GDM or overweight (mean difference = −10.27, 95% CI = −90.17 to 69.63, *p* = 0.801). The subgroup analysis revealed that probiotic intake by women with GDM decreased newborn birth weight, whereas probiotic intake by obese pregnant women increased newborn birth weight. Thus, no evidence indicates that probiotic intake by pregnant women with GDM or overweight can control newborn birth weight.

## 1. Introduction

Childhood obesity is currently one of the most severe public health concerns, and it attracts extensive attention worldwide. It is associated with a broad spectrum of adverse health outcomes. In 2016, the World Health Organization estimated that 41 million newborns and young children were overweight or obese, which is still increasing. High birth weight is associated with a two-fold higher risk of obesity in both sexes and type 2 diabetes, particularly in young male adults [1,2,3,4,5,6]. Maternal obesity and diabetes were the most potent predictors of childhood obesity [7,8,9,10], as women with obesity and gestational diabetes mellitus (GDM) have a 1.73- and 2.19-fold higher risk, respectively, of having newborns with high birth weights compared with that of pregnant women with a healthy weight [11,12,13,14,15].

To prevent transgenerational obesity, several randomized controlled trials (RCTs) focusing on lifestyle modifications and weight control during pregnancy have been conducted [16,17,18,19]. A review and meta-analysis by Thangaratinam et al. included 44 RCTs that examined lifestyle interventions during pregnancy [20]. The study concluded that exercise alone slightly reduced the birth weight of newborns. Diet-based and mixed interventions did not affect birth weight. To date, several intervention strategies have been used, but an effective and robust method to stop obesity transmission from mothers to their offspring has not been established [18,19,20,21,22,23,24,25].

The need to break the vicious cycle of obesity across generations is urgent [26,27,28,29]. One hypothesis is that obesity is transmitted through microbes from mothers with diabetes or obesity to their newborns, which means that the microbes may be directly transmitted from mothers through amniotic fluid, vaginal delivery, and placenta to the newborn and may affect the intestinal microbiome establishment in newborns [30]. Besides, high pre-pregnancy BMI and excessive weight gain during pregnancy are associated with abnormal maternal gut microbiota composition [31,32]. Therefore, maternal gut microbiota differs between women with obesity and those with a healthy weight in the latter half of pregnancy, and these differences are considered associated with increased neonatal birth weight. Studies have demonstrated that regularly consuming probiotics is beneficial in modulating gut microbiota composition, which affects the levels of microbe-derived plasma endotoxin. This may decrease the gut translocation of bacteria-derived products across the intestinal mucosa, which could contribute to systemic and placental inflammation and insulin resistance [30]. Recent hypotheses suggest that gut microbiota can control weight and energy metabolism [33]. In addition, patients with GDM present with specific vaginal and intestinal microbiome compositions, and the intestinal microbiome compositions are less diverse than those found in non-GDM mothers [34]. These microbiota alterations may be directly transmitted from mothers to the newborn gut and correlated with later microbiota colonization. It suggests that microbiota specific to maternal diabetes rather than to obesity may emerge in the newborn as a separate signature, which elicits additional risk factors for newborn health. A study on the early newborn microbiome and childhood obesity showed that despite a reduction in Bacteroidetes phylum, an early increase in *Bacteroides fragilis* species and a decrease in the genus *Bifidobacterium* were associated with increased BMI in later childhood [35]. Furthermore, probiotic supplements are associated with beneficial changes in breast milk [13,36] and reduce excessive weight gain in offspring from the fetal stage to 24–48 months of age [37].

Probiotics may effectively manipulate the human gut microbial composition and function to reduce the adverse metabolic effects associated with pathogenic microbial communities. Thus, the benefits of alternating microbes might include a reduction in oxidative stress and inflammation, reduction in intestinal permeability, and increased secretion of incretins [38]. Although numerous meta-analysis studies have focused on the influences of probiotic supplementation on the pregnant women’s metabolic profiles [39,40,41,42], none of them have emphasized its role in modifying microbiota alterations among pregnant women with GDM or obesity and its association with newborn birth weight. Studies on probiotic-based interventions in pregnant women have reported inconsistent results, whereas some studies have discovered borderline significant effects on birth weight [43]; other studies have reported the opposite result [44]. Moreover, these RCTs had limited sample sizes, making the pooling of summary results necessary. Investigating the overall effect of probiotic intervention during pregnancy on preventing childhood obesity could provide insight into future interventional studies. Therefore, this meta-analysis aimed to compare the interventional effect of probiotics on newborn birth weight in pregnant women with GDM or overweight/obesity.

## 2. Materials and Methods 

### 2.1. Search Strategy and Inclusion Criteria

An electronic literature search was conducted on PubMed, Cochrane Library, EMBASE, and Web of Science for relevant articles up to 18 December 2019. RCTs involving pregnant women with GDM or overweight were included [(probiotics OR synbiotics) AND (pregnancy OR “Gestational Diabetes Mellitus”)], [(probiotics OR synbiotics) AND (pregnancy OR overweight)] plus the Medical Subject Headings and related terms. The definitions of overweight (BMI greater than or equal to 25) and obesity (BMI greater than or equal to 30) are according to the World Health Organization. Furthermore, the reference lists of eligible studies were searched manually. The search was not restricted by language. The study protocol was registered to the PROSPERO database (registration number CRD42019138887).

All the selected studies met the following eligibility criteria: (1) Pregnant women with body mass index (BMI) ≥ 25 kg/m^2^ or diagnosed as having GDM; (2) RCTs comparing probiotic or synbiotic agents with a placebo in which probiotics were taken during the pregnancy period; (3) participant age > 18 years; (4) gestational age < 34 weeks; and (5) outcomes of newborn birth weight provided.

Studies were excluded if they met any of the following criteria: (1) included pregnant women with BMI < 25 kg/m^2^ or without GDM diagnosis; (2) were experimental trials on animals or nonhumans; (3) were non-RCT studies; (4) analyzed probiotics in conjunction with other GDM therapies in the same intervention group; (5) were abstracts, case reports, expert opinions, reviews, letters, or editorials; and (6) lacked sufficient data or did not meet the inclusion criteria.

### 2.2. Data Extraction and Quality Assessment

Two reviewers independently determined eligible studies. The following basic characteristics were extracted and tabulated: study (authors/year), study design, the sample size of the intervention group compared with the control, intervention period (category, dose, intervention time point, and duration of probiotic or synbiotic intake), and outcome as newborn birth weight. Regarding the two reviewers having different opinions, we will seek another author as the third person who will provide a different angle for the review.

The included studies were evaluated for bias using the Cochrane risk-of-bias (RoB 2.0) tool. Each included study was evaluated for the following biases: random sequence generation (selection bias), allocation concealment (selection bias), blinding of participants and personnel (performance bias), blinding of outcome assessment (detection bias), incomplete outcome data (attrition bias), selective reporting (reporting bias), and other bias. The reviewers’ judgment was categorized as “low risk,” “high risk,” or “unclear risk” of bias. The discrepancies were resolved after consultation and discussion with a third investigator.

### 2.3. Data Synthesis and Analysis

For statistical analyses, we used the Comprehensive Meta-Analysis software (version 3.2; Biostat). Newborn birth weight with 95% confidence intervals (CIs) of pregnant women who were overweight or had GDM in the included study was summarized. A random-effects model was used in the meta-analysis. Heterogeneity across studies was evaluated using Cochran’s Q test and I2 test. Subgroup analysis was performed to investigate the potential sources of heterogeneity among pregnant women with overweight/obesity compared with GDM. The Egger’s linear regression test and Begg’s funnel plot were used to determine potential publication bias. The *p* values for pooled results were two-tailed, and *p* < 0.05 was regarded as statistically significant.

## 3. Results

### 3.1. Search Results and Study Eligibility

**Figure 1:**
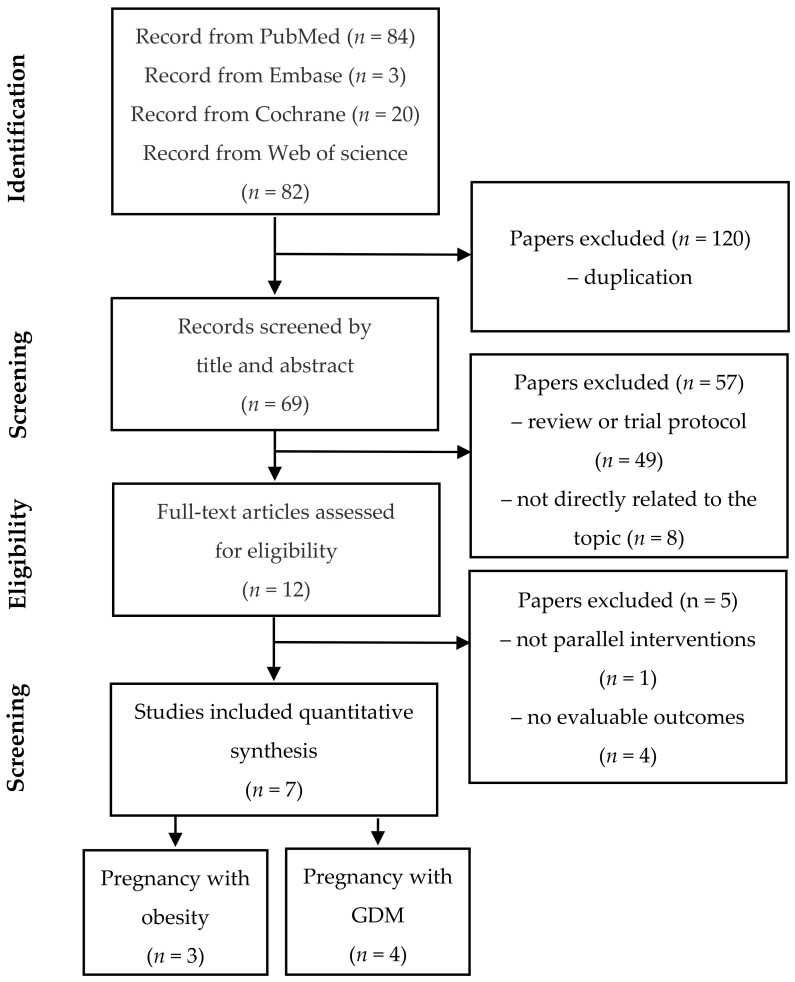
Flowchart of the study selection procedure.

A total of 189 citations were identified during the initial database search based on the predefined inclusion and exclusion criteria. Then, 182 articles were excluded for various reasons, leaving seven eligible articles involving 1093 participants, including 540 participants in the intervention group who took probiotics or synbiotics and 553 participants in the control group took a placebo. The PRISMA flowchart (Figure 1) describes the study selection process and the reasons for excluding studies.

### 3.2. Description of Selected Trials and Study Characteristics

The meta-analysis included 7 RCTs published between 2014 and 2019, and their characteristics are listed in Table 1. 4 trials [38,43,45,46] within these 7 RCTs involved probiotic intake by pregnant women with GDM. The probiotic composition varied among these four studies and included intervention strains such as *Lactobacillus salivarius* [46], *Lactobacillus acidophilus* [42,44,46], *Lactobacillus casei* [38,43], and *Bifidobacterium bifidum* [38,43,45]. One trial [43] among them also involved the use of insulin as a synbiotic supplement. The other three trials [44,47,48] within these 7 RCTs included pregnant women with overweight, and the intervention strains used were *Lactobacillus salivarius* [48], *Lactobacillus rhamnosus* [44,47], and *Bifidobacterium animalis subsp. lactis* [44,47]. The intervention duration ranged from 4 to 6 weeks in the GDM group, and that in the overweight group was 4 weeks [48] or from enrollment until giving birth [44,47]. Of the seven trials included, two were conducted in Ireland, two were conducted in Iran, and one each was conducted in Thailand, Australia, and New Zealand. The age of the pregnant women ranged from 18 to 45 years. GDM was diagnosed at 24–28 weeks in GDM groups. 3 [38,43,46] in 4 GDM studies, the average BMI of pregnant women was 28.6 ± 4.47 kg/m^2^, which meets the definition of overweight; one study [45] provided only participants’ pre-pregnancy BMI. In 3 trials of the overweight group, the average BMI of two studies [44,48] was greater than or equal to 30, which met the definition of obesity. The other one [47] was >25 kg/m^2^ during enrollment. All participants were randomly assigned to receive daily probiotics, synbiotics, or the placebo, and the daily probiotic consumption varied from 10^9^ colony-forming units (CFU)/*g* to 6.5 × 10^9^ CFU/*g* per day. We focused on the postinterventional outcome of newborn birth weight.

**Figure 2:**
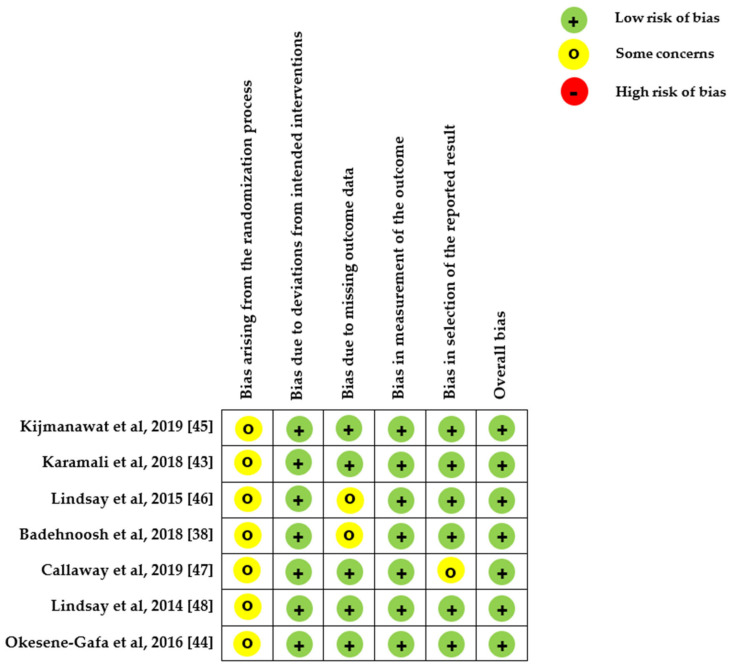
Quality assessment of the included studies conducted using RoB 2.0.

All the included studies had a low risk of bias; the risk-of-bias analysis indicated generally good methodological quality (Figure 2). Meta-regression revealed no association between newborn birth weight and study characteristics, such as the study country’s latitude.

### 3.3. Effect of Probiotics on Newborn Birth Weight in Pregnant Women with GDM or Overweight

**Figure 3:**
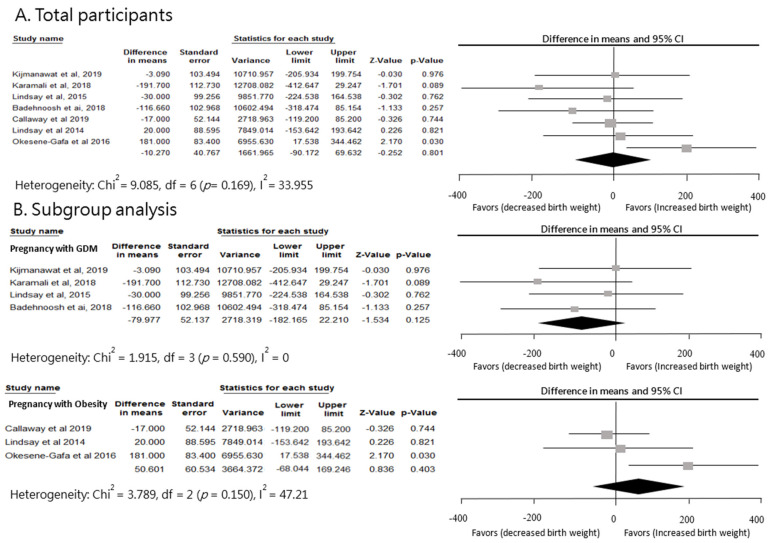
Effect of probiotic supplementation on newborn birth weight (g) in pregnant women with GDM or obesity.

A pooled analysis was performed on a total of 7 studies, but limited differences were found between probiotic and control groups for newborn birth weight (standardized mean difference = −10.27, 95% CI = −90.17 to 69.63, *p* = 0.80; Figure 3). A subgroup analysis of the efficacy of probiotics was conducted; however, the results indicated no significant difference (*p* = 0.1). Furthermore, the subgroup analyses of the GDM and overweight groups were performed. The results for newborn birth weight in the four trials of GDM groups showed a decreasing tendency of birth weight (standardized mean difference = −79.98, 95% CI = −182.17 to 22.21, *p* = 0.13). In contrast, three trials on overweight women revealed an increasing tendency of birth weight (standardized mean difference = 50.60, 95% CI = −68.04 to 169.25, *p* = 0.40). However, no clear significant difference compared with the placebo group was observed. The heterogeneity across studies in the current meta-analysis was not significant (*p*-value of Q test = 0.17; I^2^ = 33.96%).

### 3.4. Publication Bias

**Figure 4:**
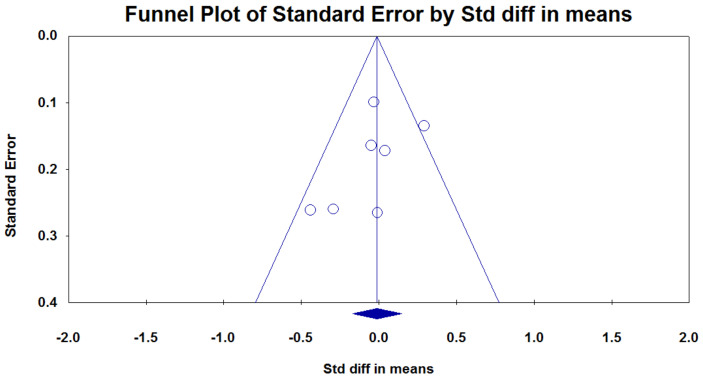
Funnel plots of publication bias: The funnel plot shows the observed mean differences (on the x-axis) against standard errors (on the y-axis). In the absence of publication bias, the plotted points form a funnel shape.

No clear evidence of publication bias was detected, and the results did not show any significant asymmetry (*p* = 0.60; Figure 4.) in Egger’s linear regression test and Begg’s funnel plot. Furthermore, we confirmed the robustness of our findings by performing a leave-one-out sensitivity analysis.

## 4. Discussion

This meta-analysis aimed to determine the association between probiotic intake and newborn birth weight of pregnant women with GDM or overweight. These results indicated that probiotic supplement interventions had limited effects on newborn birth weight. For newborn birth weight, we observed a decreasing tendency in women with GDM but an increasing tendency in pregnant women who had obesity but not GDM. Even though numerous studies have been conducted on the effects of probiotic intake during pregnancy [13,49,50,51], to our knowledge, this is the first meta-analysis to focus on the effect of probiotic intake by pregnant women with GDM or overweight on newborn birth weight. Despite current evidence supporting the microbial transmission of obesity from mothers to newborns [52], our study found that a general probiotic supplement by pregnant women with GDM or overweight might be inadequate for sufficiently influencing birth weight, revealing that probiotic intervention during pregnancy had limited disruptive effect on the vicious cycle.

Among the studies included in our systemic review, the potential mechanism of probiotics for regulating bodyweight might be due to 1. Increase epithelial cell adhesion molecules and reduce intestinal permeability, reducing systemic inflammation and insulin resistance [53]. 2. Promote the production of short-chain fatty acids (e.g., propionic acid and butyric acid), which results in the secretion of glucagon-like peptide 1 [54]. Glucagon-like peptide 1 is an incretin hormone that stimulates insulin secretion and delays gastric emptying, thus improving glucose levels [55].

The study participants included in our meta-analysis mostly enrolled in the studies in the second trimester, and the probiotics for intervention are genus *Bifidobacterium* and *Lactobacillus*. However, the studies have reported that probiotic intervention on newborn birth weight varies [55]. Karamali et al. [43] and Badehnoosh et al. [38] demonstrated the favorable effects of probiotic supplementation (three strains of bacteria: *L. acidophilus, L. casei,* and *B. bifidum strain*) for 6 weeks on newborn birth weight in pregnant women with GDM. However, Kijmanawat et al. [45] showed that probiotic supplements (two strains of bacteria: *L. acidophilus* and *B. bifidum*) had no favorable effects on newborn birth weight when given for 4 weeks to pregnant women with diet-controlled GDM. Studies have suggested that no positive outcomes result from the daily use of probiotic supplements with two strains of bacteria *(L. rhamnosus* and *B. animalis subspecies lactis*) by pregnant women with BMI > 25 kg/m^2^ from <20 weeks’ gestation until birth [47,48] or with one strain of bacteria (*L. salivarius*) by pregnant women with BMI > 30 kg/m^2^ for 4 weeks from <20 weeks’ gestation to give birth [47,48]. In addition, Lindsay et al. [46] showed that probiotic supplementation (one bacterial strain: *L. salivarius*) had no favorable effects on newborn birth weight when given to women with GDM for 4–6 weeks. Okesene-Gafa et al. [44] conducted a study about daily use of supplements with two bacterial strains (*L. rhamnosus* and *B. animalis subspecies lactis*) by pregnant women with BMI > 30 kg/m^2^ from 12–17 weeks’ gestation until birth increased newborn birth weight. This lack of a favorable effect may be attributable to these supplementations with only one or two probiotic strains and the duration of treatment for less than 6 weeks.

Previous studies tend to use multistrain probiotics because a greater number of strains means more opportunities for success, a broader efficacy range, and the possibility of an additive effect or even a synergistic effect [56,57]. As done in our study, a comparison of three probiotics with a placebo is more effective on newborn birth weight than a comparison of one or two probiotics with a placebo. Moreover, the intervention duration and critical period of probiotic intake may have resulted in heterogeneity. Lindsay et al. showed that a long administration period might be necessary for any probiotic effects to be observed in pregnant women with obesity [48]. Similar to our study, their study indicated that the positive effects of probiotic supplements on newborn birth weight are only observed after treatment for 6 weeks, not 4 weeks [48]. Besides, fetal excessive birth weight is a common adverse infant outcome of GDM. The prevention of GDM might reduce the concomitant risk of high newborn birth weight. A recent study of dietary interventions, lifestyle changes, and probiotic supplements to prevent GDM concluded that intake or exercise alone as interventions may not achieve positive results, whereas a combination of diet and lifestyle interventions may show better efficacy in reducing GDM prevalence [41]. However, the studies we included only investigated probiotic supplement interventions, not a combination of diet and lifestyle interventions. Therefore, it may limit the impact on GDM prevention and has little influence on newborn birth weight.

A study has shown that maternal obesity is a stronger predictor of a large-for-gestational-age infant than maternal hyperglycemia [58]. A large prospective study found that the upper quartile of maternal BMI was responsible for 23% of high newborn birthweight, while GDM accounted for 3.8% in Spain [59]. Those findings might explain the results of our subgroup analysis that the probiotic intervention has a positive effect on slightly decreasing newborn birth weight in the GDM group, but in the overweight group, maternal BMI still plays a crucial role in newborn birth weight.

This study had a few limitations. First, the duration of probiotic intake in the GDM group ranged from 4 to 6 weeks, which could be a possible heterogeneity source. Our findings suggest that further long-term RCTs conducted over at least 6 weeks are needed. Second, variation in the probiotics used may have increased heterogeneity among the studies. Standardized probiotic strains must be used, and participants must be appropriately stratified to identify the most efficacious strains and the extent of birth weight alternation due to probiotic supplementation. Third, the additional data on pregnancy and birth outcomes are insufficient for analyzing other possible factors influencing probiotics intake.

Further data related to pregnancy and birth outcomes should be included for additional analysis, including characteristics of mothers, such as pre-pregnancy and pregnancy body weight, BMI, age, smoking habits, insulin sensitivity test results, and delivery type, as well as the gestational age at birth and presence of neonatal jaundice or hyperglycemia. Moreover, children must follow up in the long term to capture their health profile. Finally, the relatively limited number of included studies also resulted in some bias.

## 5. Conclusions

Probiotic supplements did not considerably affect newborn birth weight in pregnant women with GDM or overweight. This meta-analysis provides essential guidance for ongoing research on probiotic supplementation protocols in pregnant women, with a more personalized probiotic intervention and exploration of the critical period of the intervention.

## Figures and Tables

**Table 1:** Characteristics of the included studies.

Study	Study Design	Country	Subjects	GA, Weeks	Age, Years	Regimen of Intervention	BW in Interventions, Mean ± SD	BW in Controls, Mean± SD	Adjustments
Kijmanawa 2019 [45]	Db-RCT	Thailand	diet-controlled GDM	24–28	18–45	I: *L. acidophilus*, *B. bifidum.* D: 10^9^ CFU/day S/E:4 weeks	3120.4 ± 411.1 *n* = 28	3123.5 ± 369.8 *n* = 29	N/A
Karamali 2018 [43]	Db-RCT	Iran	GDM, BMI: 28.45 ± 3.4	<34	18–40	I: *L. acidophilus* strain T16 (IBRC-M10785), *L. casei* strain T2 (IBRC-M10783), *B. bifidum* strain T1 (IBRC-M10771) D: 2 × 10^9^ CFU plus 800 mg inulin/day S/E: 6 weeks	3181.6 ± 459.8 *n* = 30	3373.3 ± 412.1 *n* = 30	Maternal BMI Preterm birth
Lindsay 2015 [46]	Db-RCT	Ireland	GDM, BMI: 29.00 ± 6.23	<34	>18	I: *L. salivarius* UCC118 D:10^9^ CFU/day S/E: 4–6 weeks	3570 ± 640 *n* = 74	3600 ± 570 *n* = 75	Maternal BMI
Badehnoosh 2018 [38]	Db-RCT	Iran	GDM BMI: 28.4 ± 3.8	24–28	18–40	I: *L. acidophilus, L. casei*, *B. bifidum* D: 2 × 10^9^ CFU/day S/E: 6 weeks	3321.7 ± 443.5 *n* = 30	3438.4 ± 348.4 *n* = 30	Maternal BMI Preterm birth
Callaway 2019 [47]	Db-RCT	Australia	BMI >25	<20	>18	I: *L. rhamnosus* *B. animalis subspecies lactis* D: >10^9^ CFU/day S/E: from enrollment until birth	3524 ± 540 *n* = 206	3541 ± 514 *n* = 203	Maternal BMI Preterm birth
Lindsay 2014 [48]	Db-RCT	Ireland	BMI: 30.0–39.9	<20	>18	I: *L. salivarius* UCC118 D: 10^9^ CFU/day S/E: 4 weeks	3700 ± 520 *n* = 62	3680 ± 510 *n* = 74	Maternal BMI
Okesene-Gafa 2016 [44]	2 × 2 factorial, RCT	New Zealand	BMI > 30.0	12–17	>18	I: *L. rhamnosus*, *B. animalis subspecies lactis* D: 6.5 × 10^9^ CFU/day S/E: from enrollment until birth	3685 ± 565 *n* = 110	3504 ± 672 *n* = 112	Maternal BMI Preterm birth

GA: gestational age, Db-RCT: double-blind RCT, BW: birth weight (g), Mean ± SD, I: intervention strain, D: dose, S: start of treatment, E: end of treatment. RCTs: randomized controlled trial. GDM: gestational diabetes mellitus. CFU: colony-forming units. BMI: body mass index (kg/m^2^).
